# Localized postinflammatory hyperpigmentation with abdominal wall protrusion

**DOI:** 10.1016/j.jdcr.2025.05.007

**Published:** 2025-06-04

**Authors:** Keavy Conroy, Geoffrey Hanley, Síona Ní Raghallaigh, Michael Quirke

**Affiliations:** Dermatology Department, Beaumont Hospital, Dublin, Ireland

**Keywords:** dermatomal, herpes zoster, motor complication, postinflammatory hyperpigmentation, pseudohernia

A 73-year-old Caucasian man presented with a 2-week history of right sided abdominal discomfort and swelling. Six weeks prior, he had been treated for a right sided vesicular rash by his primary care doctor. Examination revealed a diffuse, reducible nontender swelling of the right abdominal wall with overlying post inflammatory hyperpigmentation in an 11th – 12th thoracic vertebrae dermatomal distribution (Figs 1 and 2). Hyperesthesia was also noted in this area.

**Fig 1:**
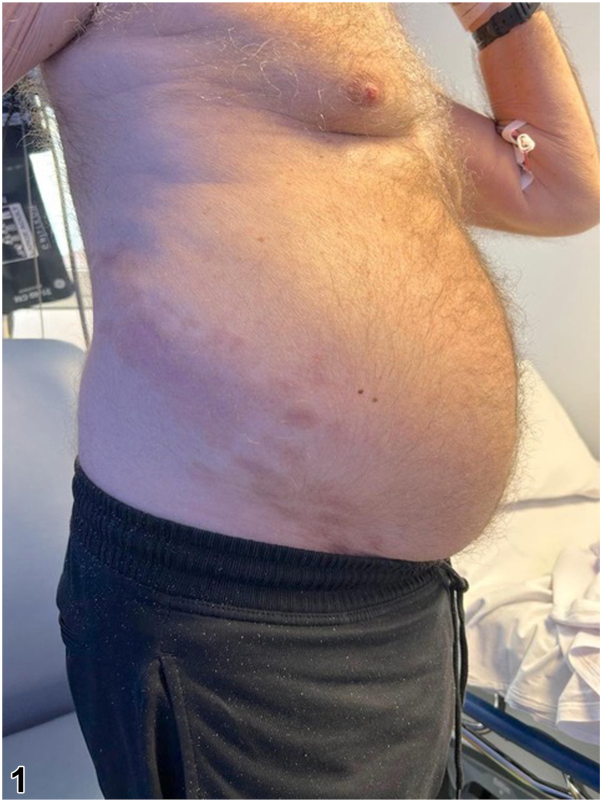

**Fig 2:**
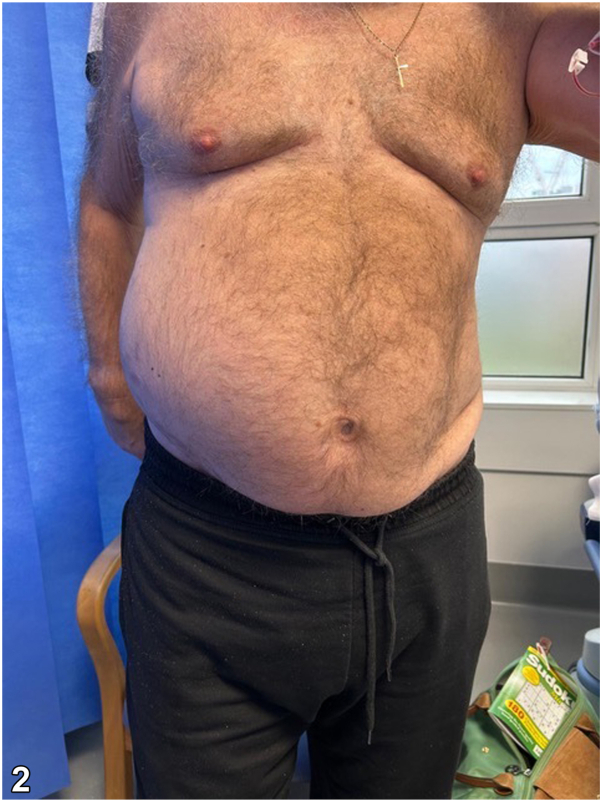

**Question 1: What is the most likely diagnosis?**

A. Spigelian hernia
B. Diastasis recti
C. Postherpetic pseudohernia
D. Erythema ab igne
E. Macrocytic lymphatic malformation

**Answers:**

A. Spigelian hernia – Incorrect. This occurs due to herniation through the Spigelian fascia comprised of transversus abdominis and internal oblique aponeuroses.
B. Diastasis recti – Incorrect. This occurs due to abnormal stretching of the linea alba, commonly associated with pregnancy, obesity, and prior abdominal surgery.
C. Postherpetic pseudohernia – Correct. A postherpetic pseudohernia is a rare motor complication of herpes zoster, occurring in 0.17% of cases.^1^ A postherpetic abdominal pseudohernia is defined by a partial protrusion of the abdominal wall caused by muscle paralysis, resulting in a unilateral swelling on the affected side. In our case, residual dermatomal postinflammatory hyperpigmentation from a recent episode of herpes zoster served as a key diagnostic clue. The onset of abdominal bulging usually occurs between 7 and 60 days following the initial cutaneous eruption.^2^ While the recognition and management of other complications of herpes zoster, such as Ramsay Hunt syndrome and herpes zoster ophthalmicus are well described, postherpetic pseudohernia remains under-recognized.
D. Erythema ab igne – Incorrect. Erythema ab igne is due to chronic exposure to heat below the threshold for thermal burn and presents with localized areas of reticulated erythema and hyperpigmentation.
E. Macrocytic lymphatic malformation – Incorrect. This is a benign cystic lesion composed of dilated lymphatic channels, which would be easily distinguished on imaging.

**Question 2: What is the most sensitive diagnostic test to help confirm the diagnosis?**

A. CT abdomen and pelvis
B. Electromyography (EMG)
C. Skin biopsy
D. Herpes serology
E. MRI abdomen

**Answers:**

A. CT abdomen and pelvis – Incorrect. A CT scan may be done to exclude a true abdominal hernia or other intra-abdominal pathology. However, it is not necessary, and is not the most sensitive test. Our patient’s imaging showed a small incidental umbilical hernia with no evidence of lateral wall defect (Fig 3).

**Fig 3:**
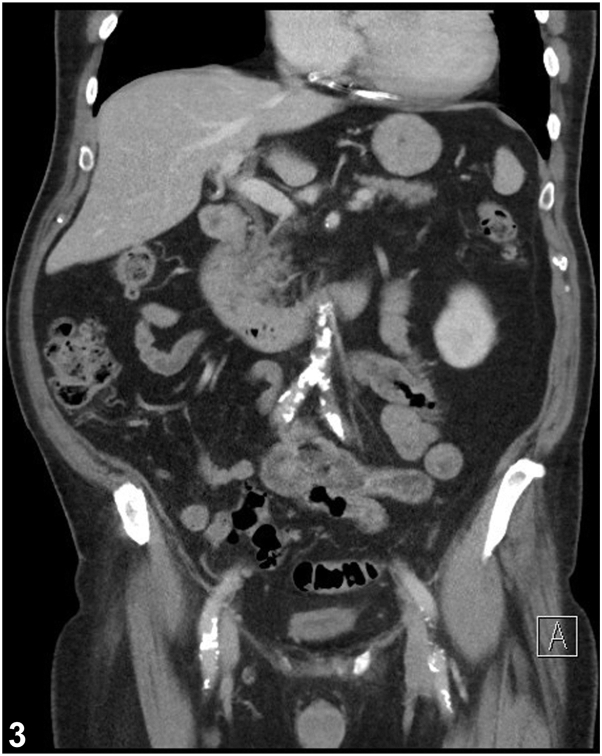
**B.** Electromyography (EMG) – Correct. Postherpetic pseudohernia is a motor complication of herpes zoster resulting from ventral nerve root denervation, leading to paralysis of the ipsilateral abdominal wall musculature^2^ EMG is a highly sensitive diagnostic modality that can detect this acute denervation, supporting the clinical diagnosis.^2^ Nerve conduction studies can also be combined with EMG to rule out other neuropathies; in cases of pseudohernia, nerve conduction studies are often normal. In this case, electrodiagnostic studies were deemed unnecessary given the close temporal correlation between rash onset and abdominal protrusion.
C. Skin biopsy – Incorrect. A biopsy would reveal patchy epidermal or dermal melanosis consistent with postinflammatory hyperpigmentation and not provide additional diagnostic information. Of note, a skin biopsy may be used to aid the diagnosis of a small fiber neuropathy; however, postherpetic pseudohernia is due to motor neuropathy.
D. Herpes serology – Incorrect. Serologic testing is not reliable for diagnosing acute herpes zoster; varicella zoster virus IgM may show delayed or cross-reactive responses, and varicella zoster virus IgG only indicates past exposure.
E. MRI abdomen – Incorrect. Although MRI abdomen may show increased T2/short-tau inversion recovery signal intensity in the abdominal wall muscles suggestive of acute denervation, it is not a first line diagnostic modality.^3^

**Question 3: What is the most appropriate management plan for this patient?**

A. Reassurance and supportive garments
B. Surgical referral
C. Antiviral therapy
D. Oral corticosteroids
E. Transcutaneous electrical nerve stimulation

**Answers:**

A. Reassurance and supportive garments – Correct. The prognosis for postherpetic pseudohernia is good with a mean recovery time of 4.9 months with conservative management.^2^ Management should include supportive garments and analgesia if required.
B. Surgical referral – Incorrect. A pseudohernia lacks a fascial defect and results from muscle weakness due to nerve dysfunction. Therefore it does not require surgical intervention. Enhanced recognition of this rare complication amongst dermatologists can prevent unnecessary investigations and surgical referral.
C. Antiviral therapy – Incorrect. Antiviral therapy for herpes zoster is most effective if started within 72 hours after the onset of acute cutaneous eruption. In our case, the patient had already been promptly treated with famciclovir by his family physician at the onset of the shingles rash. If a patient were to present acutely with cutaneous herpes zoster accompanied by pseudohernia, then there would be a role for antiviral therapy.
D. Oral corticosteroids – Incorrect. There is no evidence that oral corticosteroids improve outcomes or reduce symptom duration in postherpetic pseudohernia.
E. Transcutaneous electrical nerve stimulation – Incorrect. While there are case reports of electrical stimulation used in herpes zoster radiculopathy,^4^ it is most helpful in managing sensory disturbance, as seen in postherpetic neuralgia rather than the motor weakness itself.

## Conflicts of interest

None disclosed.
